# FeTPPS Reduces Secondary Damage and Improves Neurobehavioral Functions after Traumatic Brain Injury

**DOI:** 10.3389/fnins.2017.00006

**Published:** 2017-02-07

**Authors:** Giuseppe Bruschetta, Daniela Impellizzeri, Michela Campolo, Giovanna Casili, Rosanna Di Paola, Irene Paterniti, Emanuela Esposito, Salvatore Cuzzocrea

**Affiliations:** ^1^Department of Chemical, Biological, Pharmaceutical and Environmental Sciences, University of MessinaMessina, Italy; ^2^Department of Pharmacological and Physiological Science, Saint Louis University School of MedicineSt. Louis. MO, USA

**Keywords:** reactive oxygen species, peroxynitrite, cortical impact, inflammation, apoptosis

## Abstract

Traumatic brain injury (TBI) determinate a cascade of events that rapidly lead to neuron's damage and death. We already reported that administration of FeTPPS, a 5,10,15,20-tetrakis (4-sulfonatophenyl) porphyrin iron III chloride peroxynitrite decomposition catalyst, possessed evident neuroprotective effects in a experimental model of spinal cord damage. The present study evaluated the neuroprotective property of FeTPPS in TBI, using a clinically validated model of TBI, the controlled cortical impact injury (CCI). We observe that treatment with FeTPPS (30 mg/kg, i.p.) reduced: the state of brain inflammation and the tissue hurt (histological score), myeloperoxidase activity, nitric oxide production, glial fibrillary acidic protein (GFAP) and pro-inflammatory cytokines expression and apoptosis process. Moreover, treatment with FeTPPS re-established motor-cognitive function after CCI and it resulted in a reduction of lesion volumes. Our results established that FeTPPS treatment decreases the growth of inflammatory process and the tissue injury associated with TBI. Thus our study confirmed the neuroprotective role of FeTPPS treatment on TBI.

## Introduction

Traumatic brain injury (TBI) is a common problem that lead permanent disability in patients, particularly in the developed countries (Finnie and Blumbergs, 2002). The diagnosis of TBI is characterized by cognitive, psychological and physical problems, closely related to the injury (Albensi, 2001). TBI causes death and alterations of neuronal cells through primary injury that generate molecular and cellular cascade, that determinate the secondary injury (Bramlett and Dietrich, 2007; Werner and Engelhard, 2007). TBI damage involve metabolic changes, alterations of the oxygenation process, neuroinflammation, axonal injury, glial and neuronal cell death (Donkin and Vink, 2017). The current therapies are aimed exclusive to reduce the inflammatory process and the reactive oxygen/nitrogen species (ROS/RNS) formation that induce neuronal and cerebral blood flow alteration (Hlatky et al., 2003; Ahn et al., 2004). It has been suggested that reactive oxygen species (ROS) generation is activated at the lesion area after TBI, leading to the initial production of superoxide (${\text{O}}_{2}^{-}$) and nitric oxide (NO) radicals (Schiavone and Trabace, 2016). Among oxygen radicals, superoxide anion plays a key role in the oxidative chain reaction, producing reactive oxidant. The mitochondria are the sites of ${\text{O}}_{2}^{-}$ production in brain injury as well as under normal physiological conditions. Despite the mitochondrial detoxification, overproduced oxygen radicals, once uncoupled from this defense, induce oxidative stress (Murphy, 2009; Dröse and Brandt, 2012). Mitochondria are suggested to be the site of free radical attacks, altering ATP generation that causes energy depletion and increases free radicals (Murakami et al., 1998).

In activated endothelial cells, excessive ${\text{O}}_{2}^{-}$ react with nitric oxide (NO) that generate peroxynitrite (ONOO^−^). ONOO^−^ is the major ROS, often considered as a RNS that is a trigger for the advance of oxidative damage in brain (Beckman and Koppenol, 1996; Hall et al., 2010).

Many studies determined that ONOO^−^ mediates numerous potentially destroying chemical reactions, including lipid peroxidation together with DNA damage (Blanchard-Fillion et al., 2001; Alvarez and Radi, 2003; Besson et al., 2003). Also, ONOO^−^ suffers acid-catalyzed decomposition by different pathways (Salvemini et al., 1998b; Groves, 1999). Diverse studies have reported that certain water-soluble iron (III) porphyry's are highly active ONOO^−^ decomposition catalysts, catalyzing the isomerization of ONOO^−^ almost exclusively to nitrate producing the development of oxidizing radical species and nontoxic nitrate anion (Misko et al., 1998; Salvemini et al., 1998a). We examined ONOO^−^ role in TBI using the ONOO^−^ decomposition catalyst 5,10,15,20-tetrakis (4-sulfonatophenyl) porphyrin iron III chloride (FeTPPS). This compound, in physiologically relevant conditions, catalyzes rapid isomerization of ONOO^−^ to nitrate (N${\text{O}}_{3}^{-}$), moreover its cytoprotective actions of FeTPPS were already observed in other studies (Salvemini et al., 1998a; Jensen and Riley, 2002). In addition to the ONOO^−^ decomposing role, FeTPPS demonstrated a basal superoxide dismutase effect (Khan et al., 2011). The ONOO^−^ decomposing effect increases significantly when both ${\text{O}}_{2}^{-}$ and ONOO^−^ are present, thanks to the generation of a catalytic cycle (Jensen and Riley, 2002).

Here, we would like to demonstrate, using this catalyst, that ONOO^−^ possess a main part in the modulation of the secondary mechanism associated to TBI.

## Methods

### Animals

Male adult CD1 mice (25–30 g, Envigo, Milan, Italy) were kept five per cage under an invariable 12-h light/dark cycle, at room temperature (23°C). Food and water were accessible *ad libitum*. The study was approved by the University of Messina Review Board for the care of animals. Animal care was concordant with Italian regulations on defense of animals used for experimental and other scientific purposes (D.M.116192) as well as with the EEC regulations (O. J. of E. C. L 358/1 12/18/1986).

### CCI model

TBI was induced in mice (*n* = 10 per group) by CCI. The mice were anesthetized with Ketamine + Xylazine (2.6–0.16 mg/kg of body weight, intraperitoneal i.p,) and subjected to a cortical contusion as previous described (Logan et al., 2013). In brief, a craniotomy was made in the right hemisphere, encompassing bregma and lambda, and between the sagittal suture and the coronal ridge, with a Micro motor hand piece and drill. The resulting bone flap was removed and the craniotomy enlarged further. A cortical contusion was produced on the exposed cortex using the controlled impactor device Impact OneTM Stereotaxic impactor for CCI (Leica, Milan, Italy), the impact tip was centered and lowered over the craniotomy site until it touched the dura mater. Then, the rod was retracted and the impact tip was advanced farther to produce a brain injury of moderate severity for mice (tip diameter: 4 mm; cortical contusion depth: 3 mm; impact velocity: 1.5 m/s). Immediately after injury, the skin incision was closed with nylon sutures, and 2% lidocaine jelly was applied to the lesion site to minimize any possible discomfort.

### Experimental groups

All animals were randomized in 4 groups:

- TBI + vehicle (saline): mice (*n* = 10 mice/group) were subjected to CCI and vehicle was administered at 1 and 4 h after TBI,
- TBI + FeTPPS group: mice (*n* = 10 mice/group) were subjected to CCI and FeTPPS (30 mg/kg body weight i.p.) was administered at 1 and 4 h after TBI,
- Sham + vehicle group (saline): mice (*n* = 10 mice/group) were subjected to the surgical procedures as above group (anesthesia and craniotomy) except that the impact tip was not applied and vehicle was administered at 1 and 4 h after craniotomy,
- Sham + FeTPPS group: mice (*n* = 10 mice/group) were exposed to the surgical procedures as above group except that the impact was not done and FeTPPS (30 mg/kg body weight i.p.) was injected at 1 and 4 h after craniotomy.

In other set of experiments, additional animals were used for behavioral testing: Morris water maze test (1, 2, 3, 4, and 5 days post-TBI), Elevated Plus Maze (1, 2, 3, 6, and 10 days post-injury), rotarod test, brain water content and swing test (at 24 h after TBI). At 24 h, mice were anesthetized and 1-ml blood samples were collected from mice via cardiac puncture. The samples were centrifuged to collect plasma that stored and analyzed for biochemical parameters.

The dose and the administration way of FeTPPS, were choose on previous *in vivo* study (Genovese et al., 2007). The timing of i.p. FeTPPS injection at 1 and 4 h after TBI has the purpose of prevent or reduce the final neurological deficit to get the best possible neuroprotective effect (Sullivan et al., 2011).

### Behavioural testing

#### Elevated plus maze (EPM)

Anxiety is one of the most common neurobehavioral conditions after TBI, so anxiety deficits were evaluated using Elevated Plus Maze system at 1, 2, 3, 6, and 10 days post-TBI and compared with sham mice, as previously described (Pellow et al., 1985).

#### Rotarod test

The rotarod treadmill (Accuscan, Inc., Columbus, OH, USA) was used to measure motor function and balance (Brooks and Dunnett, 2009). Each animal was placed in a neutral position on a cylinder (3 and 1 cm diameter for rats and mice, respectively) then the rod was rotated with the speed accelerated linearly from 0 to 24 rpm within 60 s, and the time spent on the rotarod was recorded automatically. The maximum score given to an animal was fixed to 60. For testing, animals were given three trials and the average score on these three trials was used as the individual rotarod score.

#### Swing test (EBST)

The elevated body swing test (EBST) allows to provide a motor asymmetry parameter, involving handling the animal by its tail and video recording the direction of the biased body swings (Zohar et al., 2011). The EBST consisted of 20 trials with the number of swings ipsilateral and contralateral to the injured hemisphere recorded and expressed in percentage to determine the biased swing activity.

#### Morris water maze test

A spatial learning and memory test was performed by the method of Morris as previous described (Maurice et al., 1994; Iancu et al., 2005).

#### Measurement of edema

At 24 h following TBI, mice were euthanized to establish brain water content, as previously reported (Ahmad et al., 2012; Badeli et al., 2016). The cortices were quickly removed and the contralateral and ipsilateral hemispheres were weighed. Each hemisphere was dried at 60°C for 72 h, and the dry weight was determined.

Water content was calculated in ipsilateral hemisphere as:

$$\begin{array}{l} \text{Water content }(\%)=(\text{wet weight}-\text{dry weight})\\ \;/\text{ wet weight}\times 100. \end{array}$$

#### Assessment of lesion volume

At 24 h after injuries, mice were euthanized and brains were frozen. The brains were sectioned in coronal sections (14 μm). Sections were stained with haematoxylin. Area of the undamaged and injured hemisphere was measured on each section using image analysis software. The hemispheric volume was obtained by summing area of each section and multiplying it by 0.5. Lesion volume (mm^3^) was expressed as difference between the uninjured and injured hemisphere volume.

#### TTC staining

The 2,3,5-triphenyltetrazolium chloride (TTC) staining technique was used to assess infarct, as previous described (Campolo et al., 2014). The brains were cut into five coronal slices of 2-mm thickness. Slices were incubated in a 2% solution of TTC at 37°C for 30 min and immersion fixed in 10% buffered formalin solution. TTC stains the viable brain tissue red while infracted tissue remains unstained. For quantification of infarcted area and volumes, the brain slices were photographed and then image analysis was performed on a personal computer with an image analysis software program using ImageJ. To compensate for the effect of brain edema the corrected infarct volume was calculated as follow:

$$\begin{array}{l} \text{Corrected infarct area}=\text{left hemisphere area}\\ \;-\;(\text{right hemisphere area}-\text{infarct area}). \end{array}$$

Values are given as mean ± SEM. The corrected total infarct volume was calculated by summing the infarct area in each slice and multiplying it by slice thickness (2 mm).

#### Histological examination

For histopathological examination, brains were processed and damaged neurons were counted. The histopathologic changes of the gray matter were scored on a 6- point scale, as previous described (Campolo et al., 2014). The scores from all the sections from each brain were averaged to give a final score for individual animal. All the histological studies were performed in a blinded mode.

#### MPO

Myeloperoxidase activity (MPO) was determined as previously described (Cuzzocrea et al., 2007). Brain tissue collected at the specified time, were homogenized in a solution containing 0.5% hexa-decyl-trimethyl-ammonium bromide dissolved in 10 mm potassium phosphate buffer (pH 7) and centrifuged for 30 min at 20 000 g at 4°C. An aliquot of the supernatant was then allowed to react with a solution of tetra-methyl-benzidine (1.6 mm) and 0.1 mm H_2_O_2_. The rate of change in absorbance was measured spectrophotometrically at 650 nm. MPO activity was expressed in units/mg protein.

#### Nitrotyrosine immunohistochemistry

A subset of mice was randomly used to evaluate protein nitrosylation. At 24 h post-TBI, mice were sacrificed and brains were paraffin embedded, 5-μm cross sections were cut and fixed to slides. The sections were incubated with rabbit anti-nitrotyrosine antibody (Upstate Biotechnology; 5 μg/mL in 2% GS/TX/BSA) and immunohistochemistry was performed as previous described (Campolo et al., 2014).

#### Western blot analysis for IκB-α, NF-κB p65, iNOS, p-JNK, Bax, and Bcl-2

Cytosolic and nuclear extracts were prepared as previously described (Crupi et al., 2013). Western blot of protein lysates from the penumbra area of injured brains was performed as previously described (Campolo et al., 2013). The filters were blocked with 1 PBS, 5% (w/v) nonfat dried milk (PM) for 1 h at room temperature and subsequently probed with specific Abs IκB-α (1/1000; Santa Cruz Biotechnology, DBA, Milan, Italy), or anti-Bax (1/500; Santa Cruz Biotechnology), or anti-Bcl-2 (1/500; Santa Cruz Biotechnology), or anti-iNOS (1/1000; Transduction) or anti-NF-κB p65 (1/1000; Santa Cruz Biotechnology), or anti-p-JNK (1/1000; Santa Cruz Biotechnology) in 1 PBS, 5% w/v nonfat dried milk, 0.1% Tween-20 (PMT) at 4°C, overnight. Membranes were incubated with peroxidase-conjugated bovine antimouse IgG secondary antibody or peroxidase-conjugated goat anti-rabbit IgG (1/2000; Jackson ImmunoResearch, West Grove, PA, USA) for 1 h at room temperature. To ascertain that blots were loaded with equal amounts of proteic lysates, they were also incubated in the presence of the antibody against β-actin protein (1/10,000 Sigma- Aldrich Corp.). Signals were detected with enhanced chemiluminescence (ECL) detection system reagent (Sigma-Aldrich, Saint Louis, Missouri, USA) and quantified by densitometry scanning of the X-ray films with GS-700 Imaging Densitometer (GS-700, Bio-Rad Laboratories) and a computer program (Molecular Analyst, IBM), and standardized for densitometry analysis to β-actin protein levels.

#### Measurement of nitrite/nitrate production

Nitrite/nitrate production, an indicator of NO synthesis, was measured in plasma collected 24 h after TBI as previous described (Cuzzocrea et al., 1998). Briefly, the nitrate in the sample was first reduced to nitrite by incubation with nitrate reductase (670 mU/ml) and β-nicotinamide adenine dinucleotide 3′-phosphate (NADPH) at room temperature for 3 h. The total nitrite concentration in the samples was then measured using the Griess reaction, The optical density at 550 nm was measured using ELISA microplate reader. Nitrite concentrations were calculated by comparison with OD550 of standard solutions of sodium nitrite prepared in H_2_O.

#### Immunofluorescence staining

Sections were incubated with mouse monoclonal anti-GFAP (1/100, v/vol Santa Cruz Biotechnology) antibody in a humidified chamber O/N at 37 C and the detection of GFAP was obtained as previous described (Siracusa et al., 2016). Sections were observed and photographed using a Leica DM2000 microscope (Leica).

## Materials

All compounds used in this study, except where differently specified, were purchased from Sigma-Aldrich Company Ltd. (Poole, Dorset, UK). All solutions used for *in vivo* administrations were made using nonpyrogenic saline (0.9% wt/vol NaCl; Baxter Healthcare Ltd., Thetford, Norfolk, UK).

## Statistics

Data were analyzed with Graphpad PRISM V (Graphpad Software Inc., La Jolla, CA). Swing activity, time on platform, time spent on open armentries, and escape latency results were analyzed using two factor repeated measures analysis of variance (RM ANOVA, group × time). Physiological variables, time in the target quadrant, cross platform, edema, infarct area, lesion volume, MPO, NO, and densitometric analysis data were analyzed by ANOVA followed by a Bonferroni *post-hoc* test for multiple comparisons. For one way ANOVA statistic test, a single “F” value indicated as variation between sample means/variation within the samples was shown. Histological score and % total tissue area were analyzed by *t*-test. For all comparisons, *p* < 0.05 was considered significant. In the experiments involving histology or immunohistochemistry, the figures shown are representative of at least three experiments performed on different experimental days on the tissue sections collected from all the animals in each group.

## Results

### Behavioral recovery and improvement

**Figure 1:**
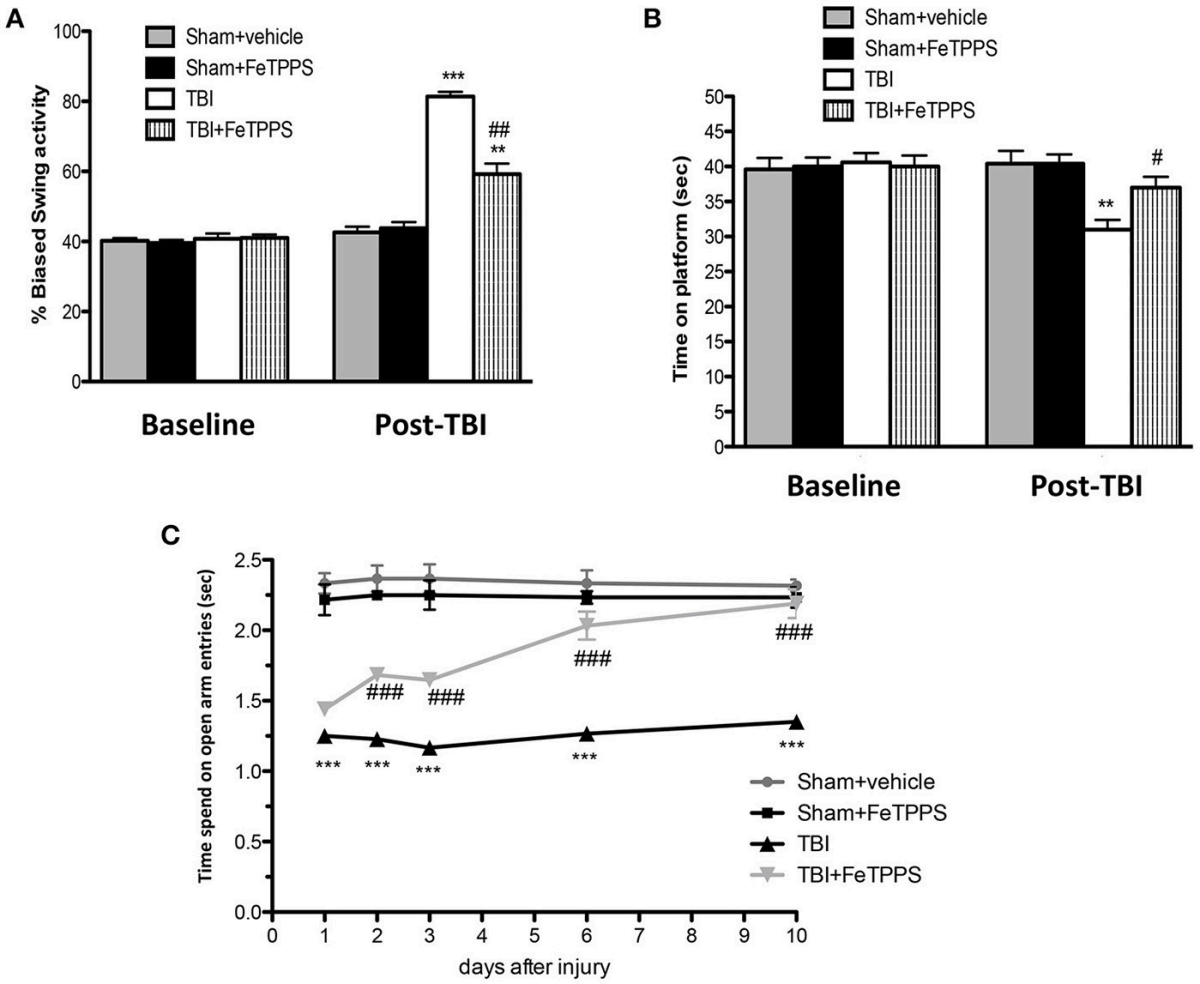
**Effect of FeTPPS on behavioral function**. FeTPPS accelerated recovery, getting an improved behavioral function. At 24 h after TBI, significant impairments were observed in motor deficits, as revealed by biased swing activity **(A)** and lower time of permanence on rotarod **(B)**. Instead, treatment with FeTPPS 1 h post-trauma significantly improved motor function, as observed in EBST **(A)** and in rotarod task **(B)**. Moreover, FeTPPS ameliorated cognitive function **(C)** as evaluated by EPM test at 1, 2, 3, 6, and 10 days after TBI. Data are expressed as mean ± SEM from *N* = 10 male CD mice for each group. A *p*-value of less than 0.05 was considered significant. ^**^p < 0.01 and ^***^p < 0.001 vs. Sham + veh; #*p* < 0.05, ##*p* < 0.01, and ###*p* < 0.001 vs. TBI + veh.

Mice injured showed behavioral deficits. Two different tests, the EBST and the Rotarod test, that are the most sensitive vestibulomotor tests (Iancu et al., 2005), were used to examine the relationship between neurological deficits in the setting of TBI. CCI-injured mice showed a series of impairments in locomotors tasks as observed in Figure 1.

First of all, to evaluate motor function impairment mice were subjected to the elevated body swing test (EBST), 24 h after TBI. Sham group had no apparent bias in swing behavior (Figure 1A), whereas FeTPPS treatment significantly ameliorated influence on the swing bias (Figure 1A) compared to TBI group (Figure 1A). Moreover, to test overall balance, motor coordination, and motor learning we performed Rotarod test. The sham-operated mice were not observed to slip from the rod at any time (Figure 1B), whereas injured mice found it difficult to walk precisely on the rod, tending to lose their balance (Figure 1B). FeTTPS treatment improve the performance of mice that remained on the rotor for longer periods (Figure 1B).

Subsequently, to assess the anxiety, a decisive component of behavioral change after brain injury, CCI injured animals were subjected to the Elevated Plus Maze (EPM) at 1, 2, 3, 6, and 10 days; also, mice were treated with FeTPPS every day, observing an improved latency compared to TBI + vehicle group (Figure 1C).

### Effects of FeTPPS on the performance after TBI in morris water maze test (MWM)

**Figure 2:**
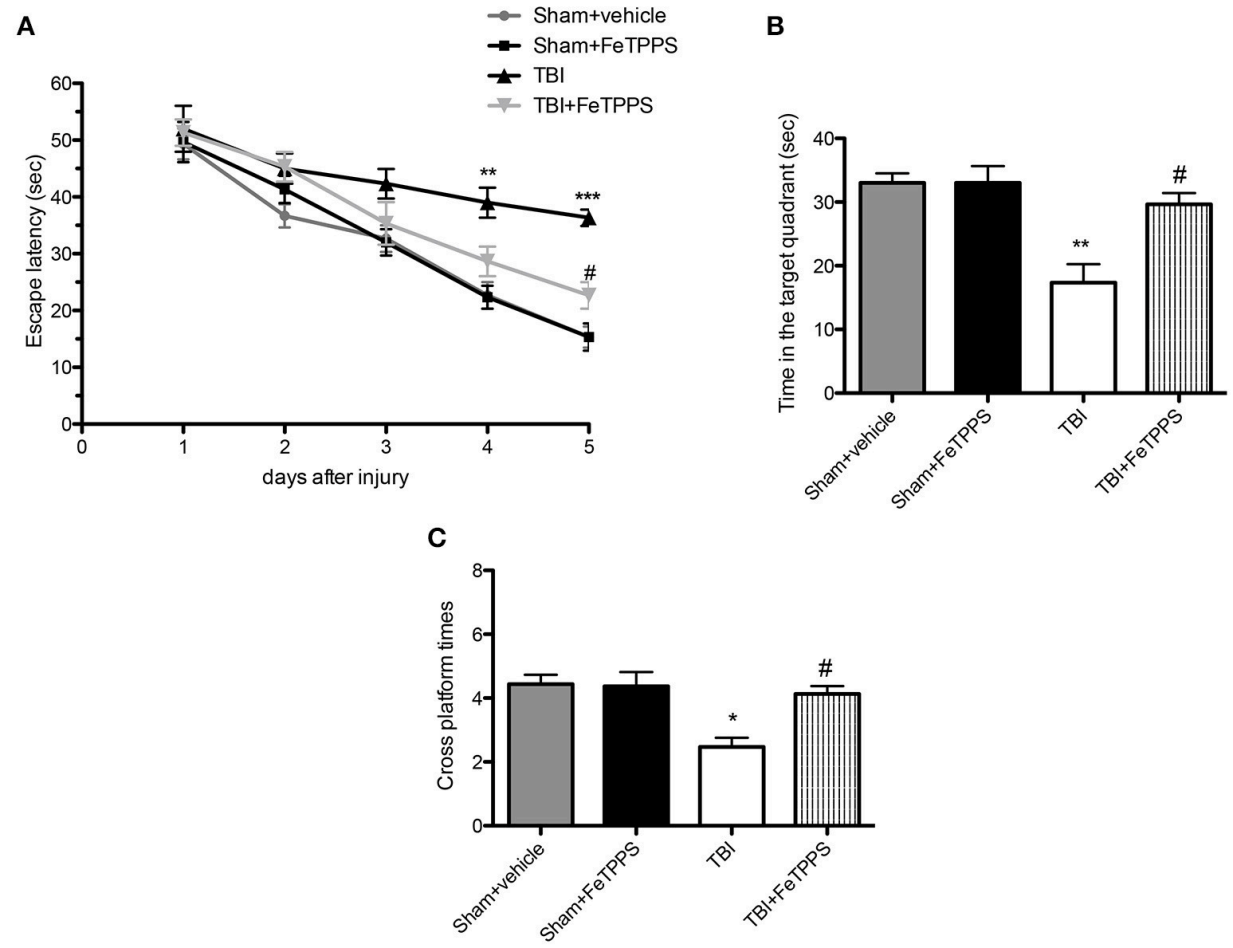
**Effect of FeTPPS treatment on the performance of Morris Water Maze (MWM) in mice after TBI**. The MWM was utilized to analyze spatial learning and memory to allow for recovery of motor deficits. Escape latency **(A)**, time in the target quadrant **(B)**, and cross platform times **(C)** were measured. Data are means ± SEM of 10 animals for each group, ^*^p < 0.05, ^**^p < 0.01, and ^***^p < 0.001 vs. Sham + veh; #*p* < 0.05 vs. TBI + veh.

In the MWM test, the TBI group exhibited longer escape latency on day 1, 2, 3, 4, and 5 (Figure 2A), compared with the sham group and sham+ FeTPPS. However, the FeTPPS treatment significantly ameliorated the effects of TBI on escape latency. Memory retention of the podium position was assessed in the spatial probe test performed on the day following the place navigation test; TBI mice spent minor time in the target quadrant respect to control groups, although the difference in cross platform times was not markedly different. The shorter swimming time in the target quadrant induced by TBI was significantly restored by FeTPPS (30 mg/Kg) (Figures 2A–C). No significant difference was noted among all the groups in swimming speed throughout the test (data were not shown).

### Decrement of edema and brain infarctions following trauma

**Figure 3:**
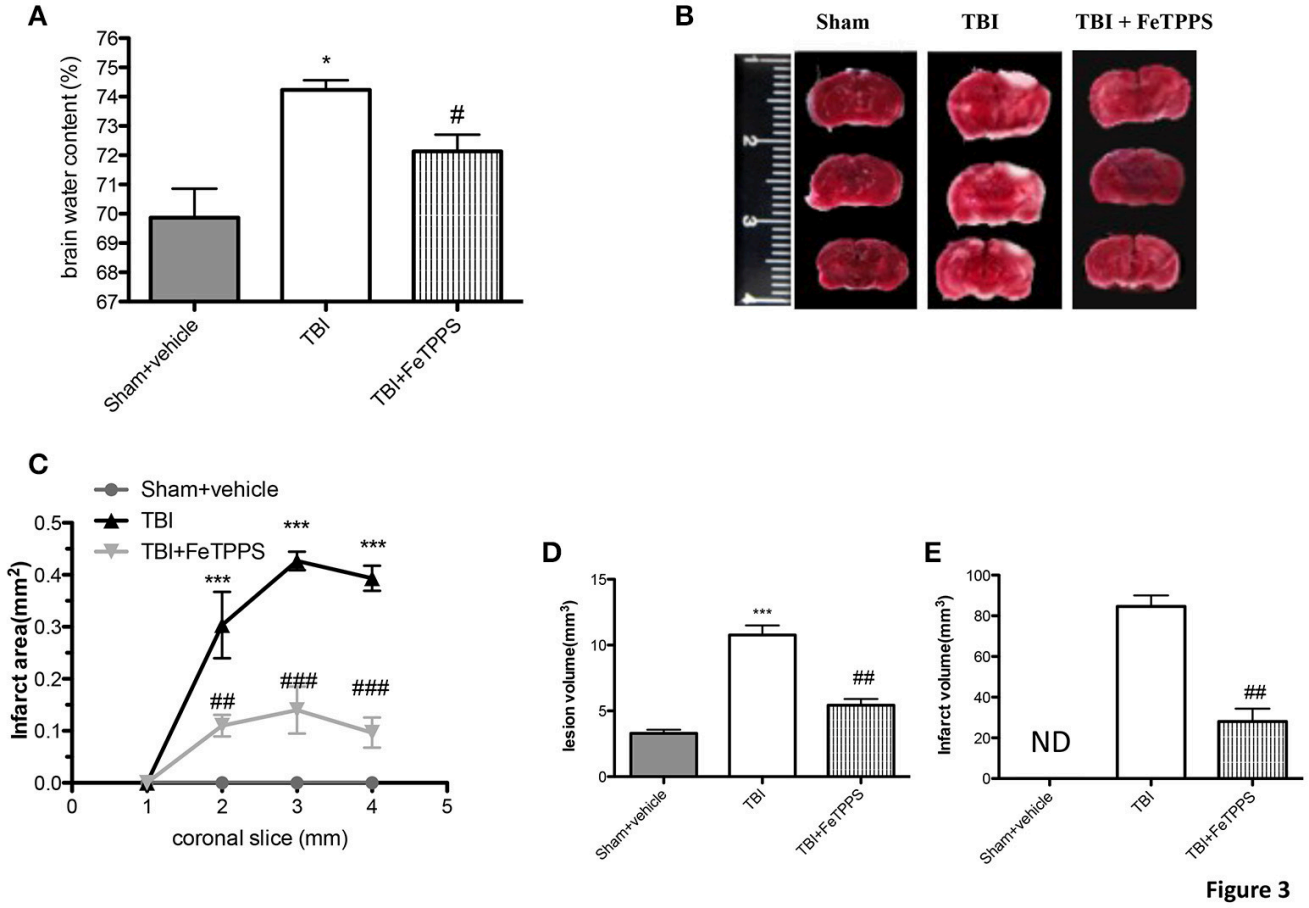
**FeTPPS reduces brain edema and infarctions following TBI**. At 24 h after trauma were observed increased levels of water content in the TBI brain **(A)**, while the treatment with FeTPPS decreased the brain edema **(A)**. Brain sections (2 mm thick) were stained with TTC at 24 h post-trauma **(B)** to show significant reduction in infarct area **(C)** and volume **(D,E)**. Lesion volume measured by hematoxylin after injury was significantly larger in CCI group vs. sham group **(E)**. ^*^p < 0.05, and ^***^p < 0.001 vs. Sham + veh; #*p* < 0.05, ##*p* < 0.01, and ###*p* < 0.001 vs. TBI + veh.

Brain water content is a sensitive measure scoring pathology associated with endothelial cell activation and dysfunction. The brain water content was significantly different between all groups with levels substantially more high in TBI-injured mice compared to sham group, as shown in Figure 3A. FeTPPS treatment at 24 h post-injury significantly reduced the increased water content in the ipsilateral brain, caused by TBI. To appraise the effects of FeTPPS on brain infarctions, we performed the 2,3,5-triphenyltetrazolium chloride (TTC) staining (Figure 3B). The infarct area (Figure 3C) and infarct volume (Figures 3D,E) were significantly reduced after treatment with FeTPPS (Figures 3C,D).

### Histological examination

**Figure 4:**
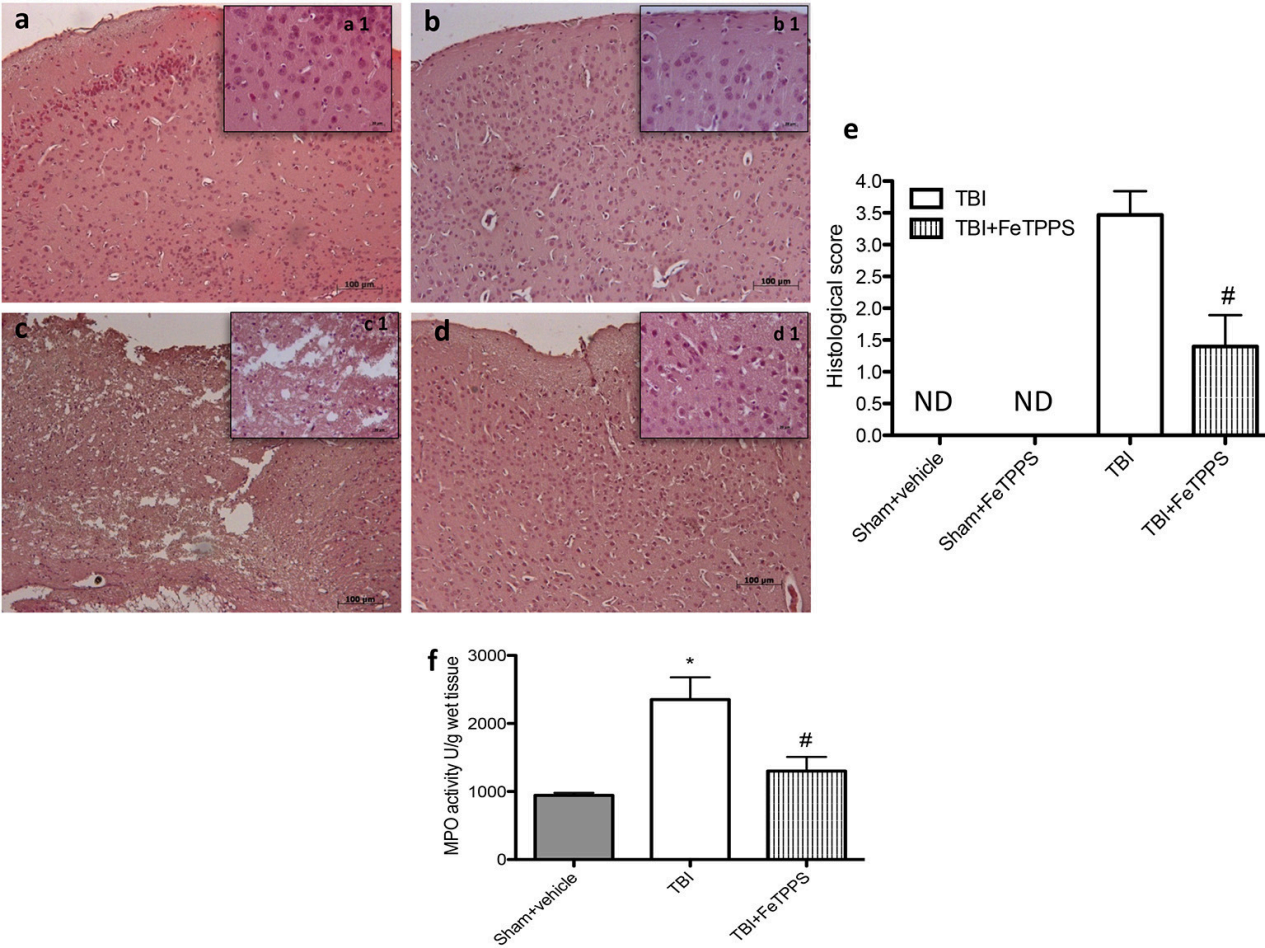
**Severity of Brain damage on histological alterations and MPO activity**. FeTPPS reduces the severity of brain trauma. No alteration in brain tissues from control mice (**a**, histological score **e**) and sham + FeTPPS (**b**, histological score **e**) was evident. On the contrary, the low-magnification images of damaged brain showed tissue disorganization and inflammation in the perilesional area at 24 h after injury (**c**, histological score **e**). A considerable protection from the TBI was apparent in the tissue collected from FeTPPS treated mice (**d**, histological score **e**). Moreover, MPO activity was significantly increased in the brain **(f)** from TBI-induced mice in comparison with sham mice. FeTPPS treatment significantly reduced the TBI-induced increase in myeloperoxidase activity. Data are means ± SEM of 10 mice for each group, ^*^p < 0.05 vs. Sham + veh; #*p* < 0.05 vs. TBI + veh.

Histological examination of the brain from TBI animals, at 24 h post-damage, showed significant tissue disorganization, inflammation in the perilesional area and white matter alteration (Figure 4c, histological score Figure 4e) compared to control (Figure 4a, histological score Figure 4e) and compared to sham animals that received FeTTPs (Figure 4b). We showed that the treatment with FeTPPS (30 mg/kg) notably reduced the degree of brain injury (Figure 4d, histological score Figure 4e). In addition, the above-mentioned histological pattern of TBI seemed to be associate with the incursion of leukocytes into the brain. Therefore, we analyzed the effects of FeTPPS on neutrophilic infiltration by measuring brain MPO activity and we observed that MPO activity was considerably elevated in the brain of mice at 24 h after trauma compared with sham-operated animals (Figure 4f). Treatment with FeTPPS attenuated neutrophilic infiltration into the brain at 24 h after injury.

### TBI effects of FeTPPS on nitrotyrosine formation and NO production

**Figure 5:**
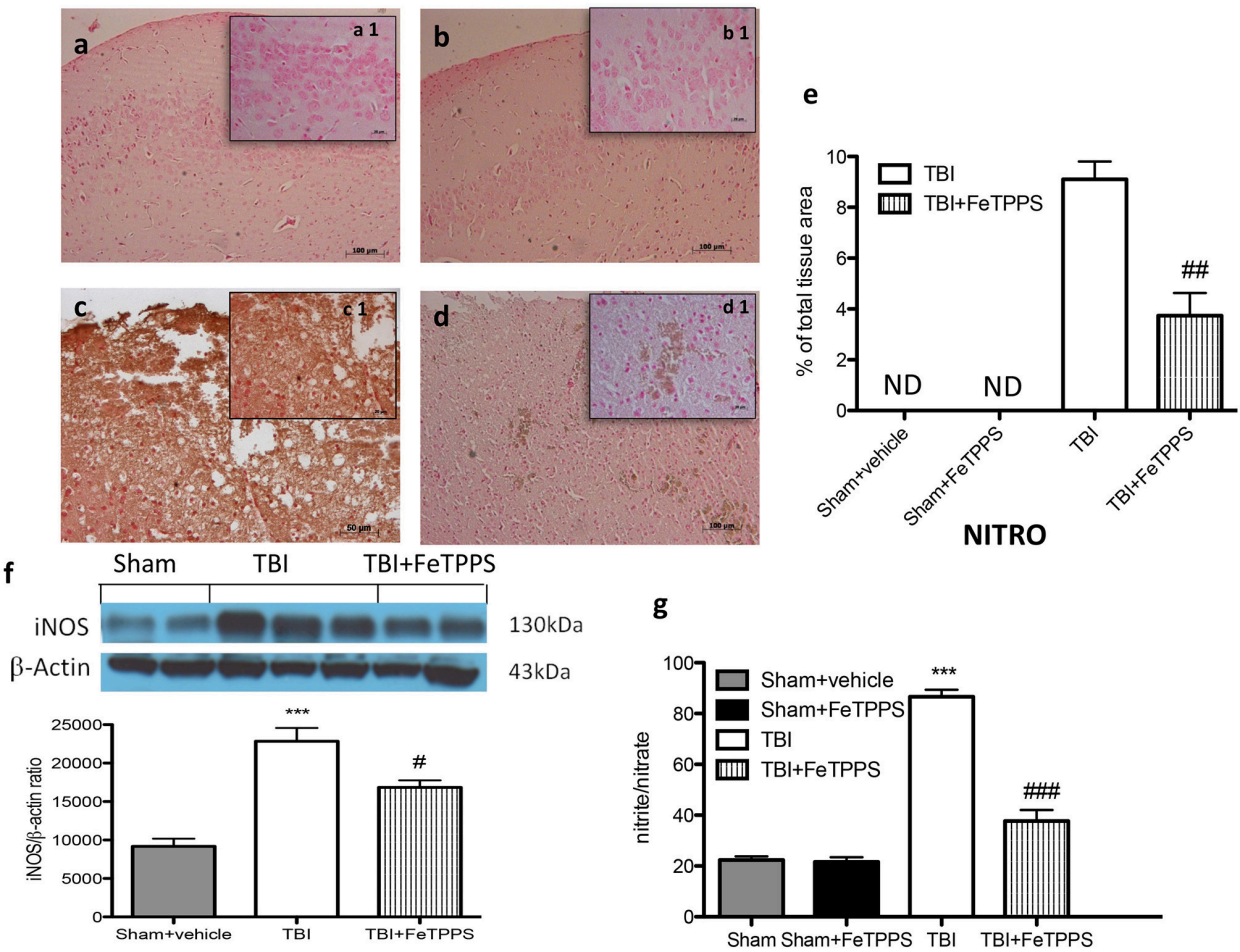
**Effects of FeTPPS on NO production after TBI**. Brain tissue section from sham mice **(a)** and sham + FeTPPS **(b)** haven't shown any positive staining for nitrotyrosine. Sections from TBI-injured mice demonstrated positive staining for nitrotyrosine **(c)**, notably reduced by FeTPPS treatment **(d)**. In addition, iNOS expression and plasma levels of nitrite/nitrate (NO_2_) were also performed. At 24 h after trauma, an important increase in the iNOS expression **(f)** and plasma levels NO_2_- were observed in brain **(g)** compared with the sham mice. FeTPPS treatment significantly reduced iNOS expression and NO_2_ plasma levels. β-actin was used as internal control. A representative blot of lysates obtained from each group is shown, and densitometry analysis of all animals is reported (*N* = 10 mice from each group). ^***^p < 0.001 vs. Sham + veh; #*p* < 0.05, ##*p* < 0.01, and ###*p* < 0.001 vs. TBI + veh; F 102.8.

To verify the localization of different reactive nitrogen species produced during TBI, nitrotyrosine was measured by immunohistochemical analysis, 24 h after brain damage. Brain sections from control mice (Figure 5a, densitometry analysis Figure 5e) and FeTPPS-treated animals (Figure 5b, densitometry analysis Figure 5e) did not stain for nitrotyrosine, while brain sections obtained from injured mice exhibited a positive staining for nitrotyrosine (Figure 5c, densitometry analysis Figure 5e), localized in inflammatory tissues. Treatment with FeTTPs significantly reduce the positive staining for nitrotyrosine (Figure 5d). To determine the role NO produced during TBI, inducible nitric oxide synthase (iNOS) expression was evaluated by western blot. An important increase in iNOS expression was observed in the brain from TBI- injured mice (Figure 5f). On the contrary, FeTPPS treatment prevented the TBI-induced iNOS expression (Figure 5f). Moreover, the analysis conducted on the plasma of all animals showed that in the mice subjected to TBI there was a significant concentration in NO_2_ compared to sham mice. The increase in plasma NO_2_ levels was significantly reduced in animals treated with FeTPPS (Figure 5g).

### Effect of FeTTPS on GFAP expression

**Figure 6:**
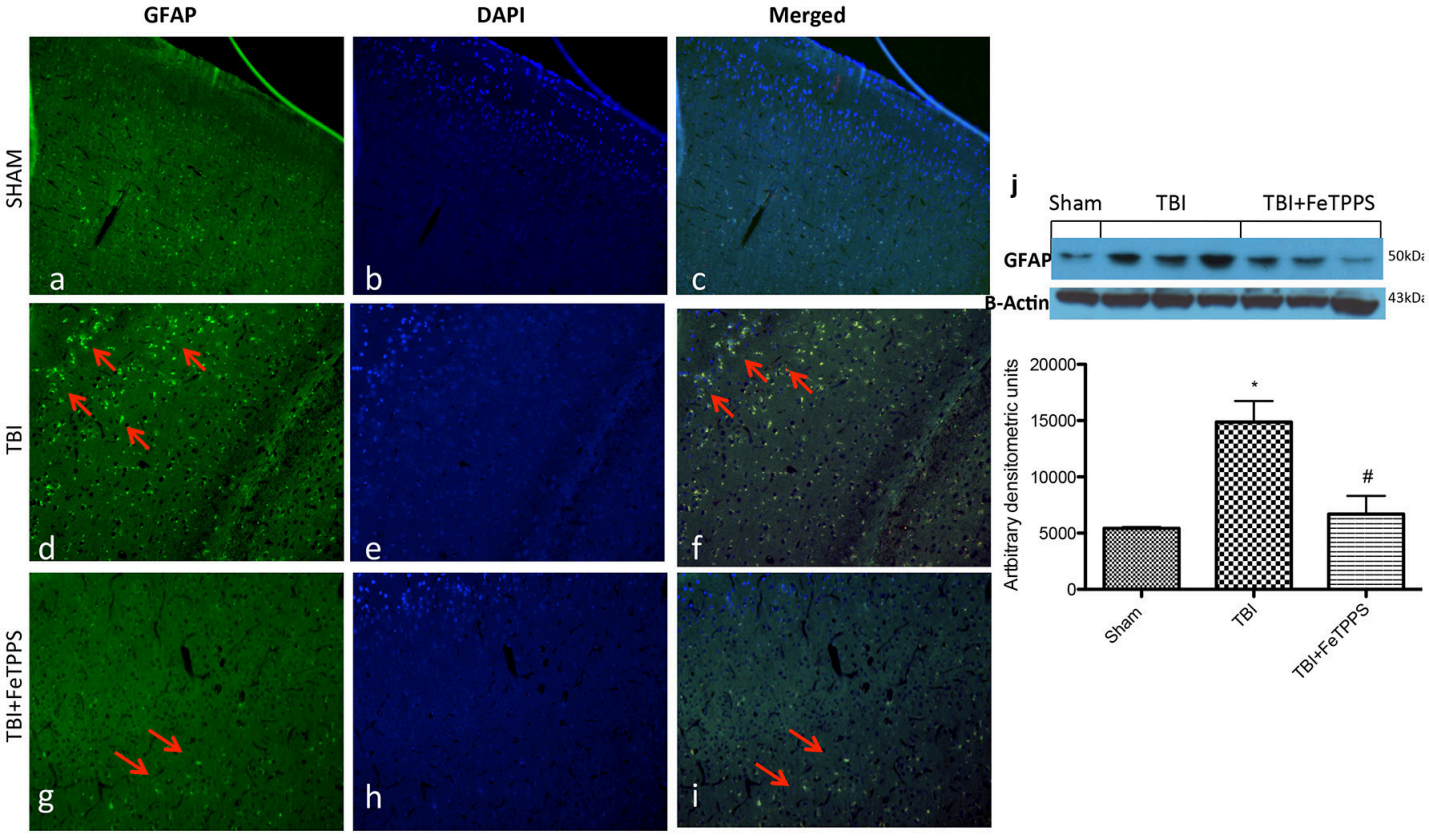
**FeTPPS reduces the GFAP immunoreactivity following TBI at 24 h post-injury**. Compared to the GFAP immunoreactivity in unaltered control tissue, significantly increased number of GFAP positive astroglial cells were detected in TBI mice **(d)**. The effect was attenuated by FeTPPS treatment **(g)**. Fluorescent photomicrographs from brain tissue stained with DAPI (blue; **b,e,h**), GFAP (green; **a,d,g**), and a merged image of DAPI and GFAP **(c,f,i)** were shown. Western blot analysis demonstrated that FeTTPS reduced GFAP expression **(j)**. ^*^p < 0.05 vs. Sham + veh;. #*p* < 0.05 vs. TBI + veh. F 13.01.

The relative fluorescence intensity of GFAP was evaluated in brain tissues. In the injured brains, a major expression for GFAP was observed in the TBI (Figure 6d); astrocytes activation was significantly lower in FeTPPS vs. TBI groups (Figure 6g). GFAP levels were basal in sham group (Figure 6a). The red arrow indicates the localization of GFAP (Figures 6c,f,i) and DAPI, staining assay was performed to reveal the cell viability (Figures 6b,e,h). Moreover GFAP expression was evaluated by western blot analysis. We clearly demonstrated that FeTPPS treatment reduces GFAP expression, that was significantly increased after TBI (Figure 6j).

### Effects of FeTPPS on NF-κB p65 pathway

**Figure 7:**
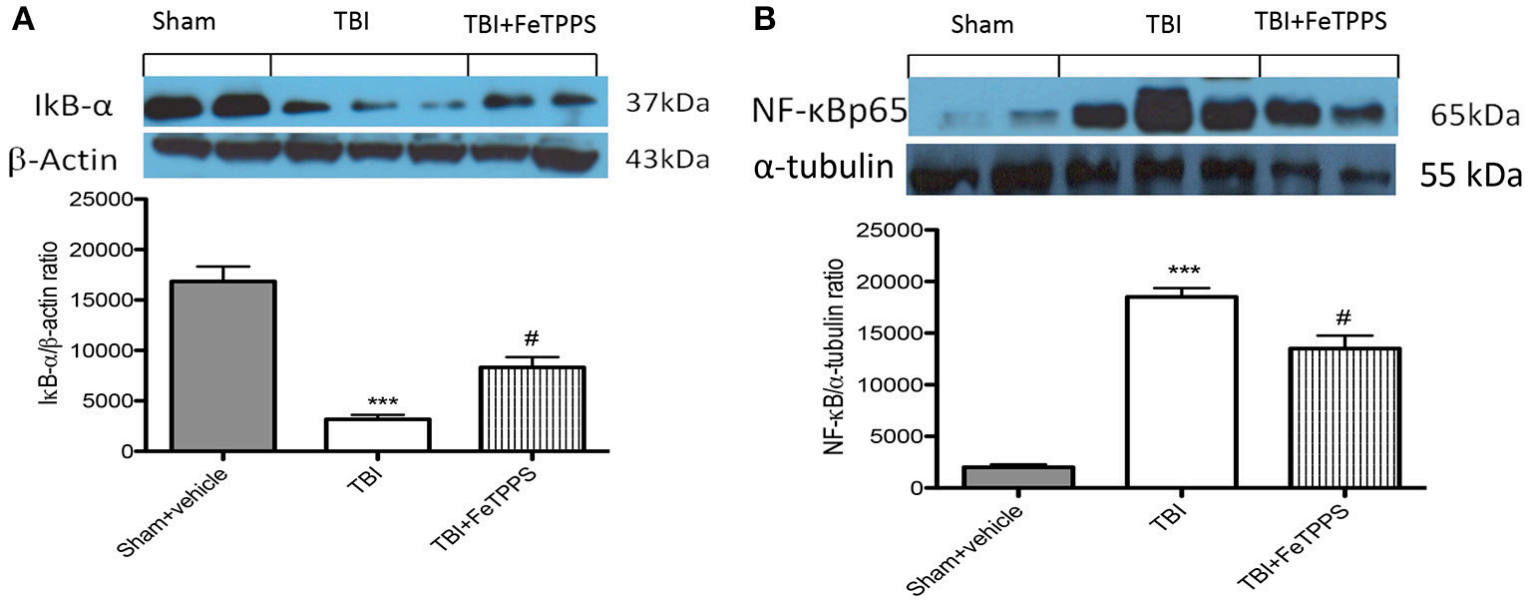
**Effect of FeTPPS on IkB-a, NF-κB p65**. IkB-α was significantly degraded in brain tissues obtained from TBI-induced mice **(A)**. FeTPPS treatment mice prevented TBI-induced IkB-α degradation **(A)**. Nuclear NF-κB p65 translocation was significantly increased in brain nuclear fractions from TBI-induced mice compared with FeTPPS treated mice **(B)**. A representative blot of lysates obtained from five animals per group is shown, and densitometry analysis of all animals is reported. ^***^p < 0.001 vs. Sham + veh;#*p* < 0.05 vs. TBI + veh; *F*-value for IkB-α 33.81; *F*-value for NF-κB p65 58.21.

We evaluated inhibitor of kappa B (IkB-α) degradation, nuclear factor kappa B (NF-κBp65) translocation and p-JNK expression by western blot analysis to investigate the cellular mechanisms whereby treatment with FeTPPS is protective in TBI. Basal expression of IkB-α was detected in brain homogenates from sham-operated mice, whereas IkB-α were substantially degradated in brain tissue collected from TBI mice (Figure 7A). FeTPPS treatment impeded TBI-induced degradation of IkB-α (Figure 7A). Moreover, NF-κB p65 translocation in the brain nuclear fractions was also considerably increased 24 h after TBI respect to control mice (Figure 7B). FeTPPS treatment appreciably reduced the expression of NF-κB p65 (Figure 7B).

### Effects of FeTPPS on MAPK expression

**Figure 8:**
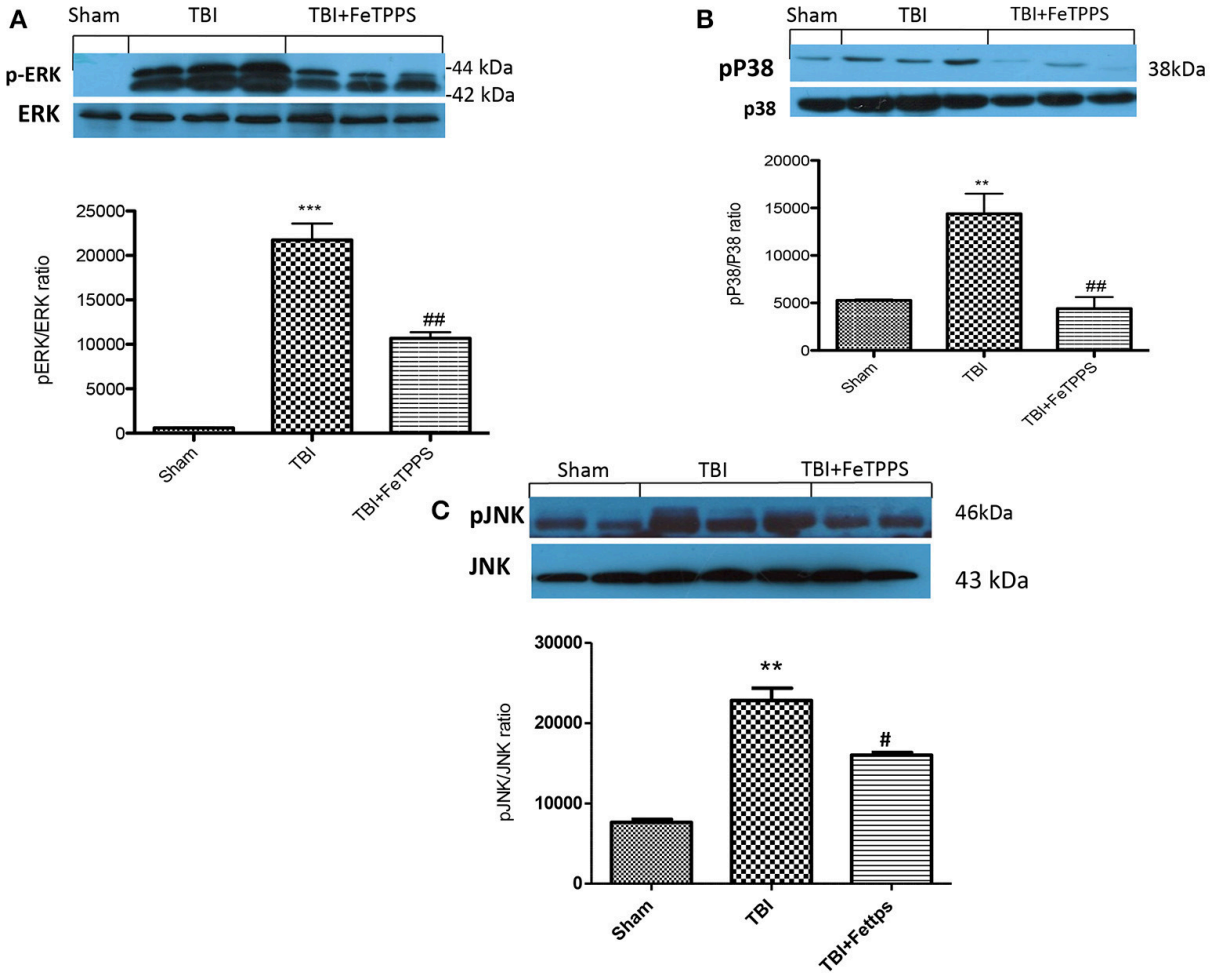
**Effect of FeTPPS on MAPKs expression**. A significant increase in p-ERK **(A)**, pP38 **(B)**, and p-JNK **(C)** were observed in brain tissues obtained from TBI mice compared with sham animals: FeTPPS restored the expression of p-ERK **(A)**, pP38 **(B)**, and p-JNK **(C)**. ^***^p < 0.001 vs. Sham + veh; ^**^p < 0.01 vs. Sham + veh; ##*p* < 0.01 vs. TBI + veh. #*p* < 0.05 vs. TBI + veh. *F*-value for p-Erk 85.83; *F*-value for p-P38 15.47; *F*-value for p-JNK 38.96.

At 24 h after TBI, the expression of MAPK, such as p-ERK, pP38, and p-JNK in brain homogenates were also investigated. Basal expression of MAPKs were detected in brain homogenates from sham-operated mice, whereas MAPKs expression were considerably increased in TBI mice (Figures 8A–C, respectively). FeTPPS treatment prevented TBI-induced MAPKs increase expression (Figures 8A–C, respectively).

### Effects of FeTPPS on apoptosis

**Figure 9:**
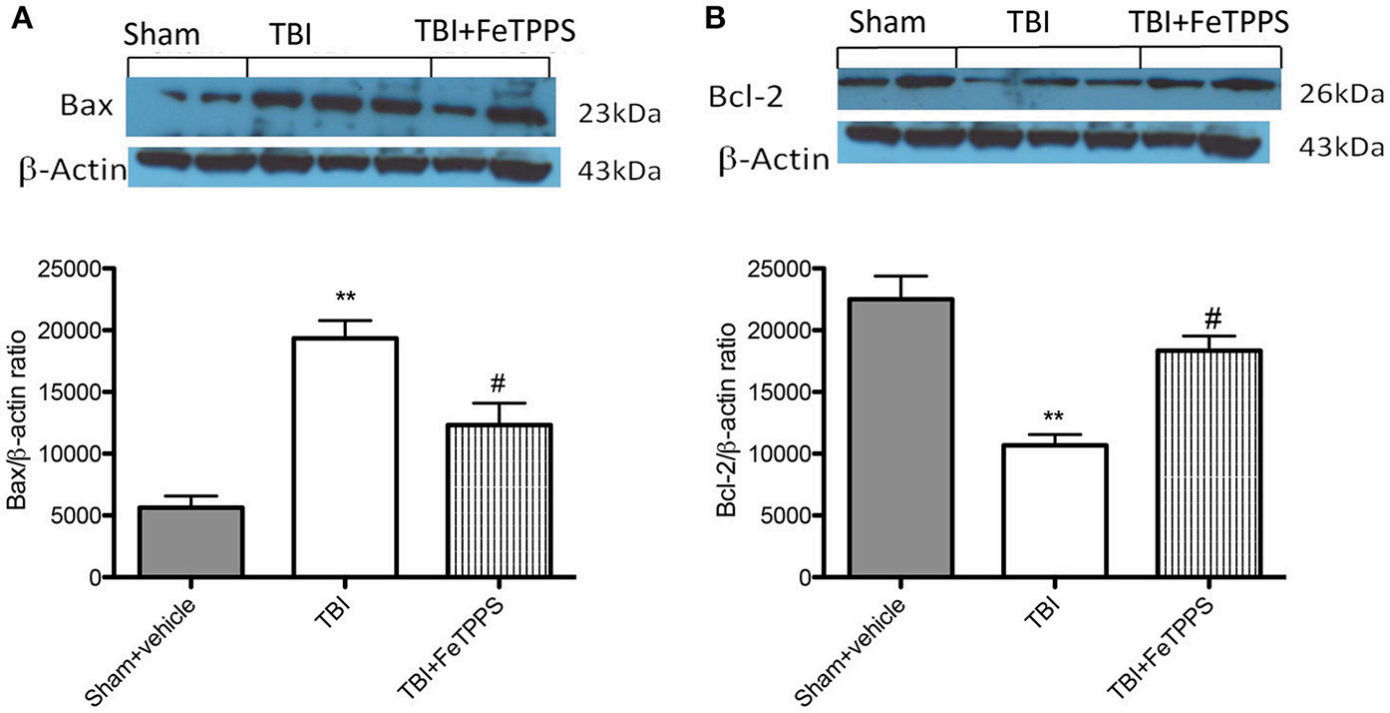
**Effects of FeTPPS on apoptotic proteins in brain tissue**. Western blot data detected a significant increase in Bax expression **(A)** in the brain tissues caused by TBI, while FeTPPS treatment significantly reduced the proapoptotic Bax expression in the brain at 24 h after injury. Moreover, Bcl-2 levels were significantly lowered in TBI-injured mice at 24 h **(B)**, but significantly restored by FeTPPS treatment. ^**^p < 0.01 vs. Sham + veh; #*p* < 0.05 vs. TBI + veh. *F*-value for Bax 45.85; *F*-value for Bcl2 26.88.

To investigate if brain trauma was correlated to apoptosis, the appearance of proteic effectors of canonical mitochondrial apoptosis, such as pro-apoptotic (Bax) proteins and anti-apoptotic (Bcl-2) proteins, was investigated by western blot analysis. Bax levels were appreciably increased in the brain from mice subjected to TBI (Figure 8A). On the contrary, FeTPPS treatment banned TBI-induced Bax expression (Figure 9A). Moreover, in the brain extract from sham mice a basal level of Bcl-2 was detected (Figure 8B). In TBI-induced mice, Bcl-2 expression was significantly reduced (Figure 9B), while FeTPPS administration determined an increase in Bcl-2 expression (Figure 9B).

## Discussion

TBI is a major problems that afflict the society with an increased incidence in young adults (Maas et al., 2008). Multiple factors affected the outcome of brain injury and currently, there aren't neuroprotective treatment strategies to get better neurological outcome after trauma. The use of *in vivo* models for TBI enhanced the comprehension of TBI pathophysiology, in particular CCI models result in an ipsilateral injury that reproduce damages similar to that observed in humans, such as cortical contusion, edema, elevated intracerebral pressure, subarachnoid hemorrhage, decreased cerebral blood flow, neuro-endocrine and metabolic dysfunctions (Morales et al., 2005). Moreover, CCI model, reproduces numerous features of brain injuries, including neuron less, motor and cognitive deficits (Colicos et al., 1996; Hoffman et al., 2008; Kline et al., 2008). The gravity of injury can be controlled by changing velocity and profundity of impact and the size of the impactor tip (Dixon et al., 1991). It is accepted that very severe trauma involves numerous pathways. Following trauma it is observed a dramatic breakdown of membrane and DNA, caused by the increased action of free radical endogenous scavenging molecules, that finally determined cell death (Bains and Hall, 2012).

It is known that TBI elicits behavioral function impairment, probably due to the over activation of glutamate receptors in the hippocampus that confounds rehabilitation and concurs to a poor quality of life. The neurological deficit was evaluated with two different tests: rotarod test that revealed an increase performance of mice treated with FeTTPS compared to TBI animals; EPM showed increased anxiety-like behavior in mice subject to TBI and this behavioral phenotype was reversed by FeTTPS administration. Improvement of learning and memory remains a significant goal of TBI research; unluckily, up to day there is no promising therapies for TBI.

The role of reactive-oxygen-induced oxidative injury in TBI was strongly observed in various studies (Benuck et al., 1992; Deng et al., 2007), that demonstrated that ROS production and inflammatory process continues for at least 1 h after damage (Cobbs et al., 1997). Through the reactive-oxygen-induced oxidative damage ONOO^−^plays important roles, it is very reactive toward biological molecules (Salvemini et al., 2006) and sufficient stable to cross cell and to contact target cells prior to becoming protonated (Crow and Beckman, 1995). A better clarification of the function of ONOO^−^ in human disease should provide a great contribution in the design of balanced therapies for pharmacological intervention. To this purpose, FeTPPS with its properties, could be represent a new therapeutic approach for treat TBI. This consideration is based on a previous study in which FeTMPS, another ONOO^−^ decomposition catalyst, decreased the expression of pro-inflammatory cytokines in a model of I/R of the gut (Cuzzocrea et al., 2000).

Moreover, we already demonstrated that FeTSPP possess anti-inflammatory and neuroprotective effects in an another disease in which ONOO^−^ is implicated in a generation of secondary cytotoxic neuronal damage during spinal cord injury (SCI) (Genovese et al., 2007).

We clearly demonstrated that FeTSPP treatment reduced the development of inflammation and tissue injury associated with spinal cord trauma similarly to a well-known anti-inflammatory agent that were used as positive control.

Nevertheless implications for the contribution of ONOO^−^ to disease are based principally on nitrotyrosine formation, that is marker of “footprint” ONOO^−^ and nitrosative stress. Thus, in this study we have observed that FeTPPS considerably reduced nitrotyrosine formation.

Moreover NO in turn control GFAP expression in astrocytes (Brahmachari et al., 2006). Though the astrocyte activation implies the production of different neurotrophins for neuronal survival, at the same time it is implicates in the inflammatory process, provoking brain and neuronal damage (Tani et al., 1996). Thus, here we also evaluated by immunofluorescence analysis the expression of GFAP, as astrocyte activation marker, and the results obtained demonstrated a high expression of GFAP after TBI compared with FeTPPS-treated mice.

The increase in oxidative stress has, as the consequence, the activation and inactivation of redox-sensitive proteins (Bowie and O'Neill, 2000). Recent studies propose that the activation of NF-κB may also be controlled by an oxidant/antioxidant balance (Haddad, 2002). NF-κB is normally located in the cytoplasm, bound to regulatory protein IκBα. Pro-inflammatory stimuli induce phosphorylation of IκB by the enzyme IκB kinase (Bowie and O'Neill, 2000) with the release of NF-κB, which is then free to translocate in the nucleus. Thus in the present study we demonstrated that FeTPPS inhibited the IκB-α degradation and NF-κB translocation demonstrating that ONOO^−^also regulated the equilibrium between proinflammatory and prosurvival roles of NF-κB. Moreover, NF-κB control the expression of inflammatory protein, such as iNOS, TNF-α and IL-1β (Salvemini and Cuzzocrea, 2002). Moreover, the inflammatory process that follow TBI, is correlated with lipid peroxidation and neutrophil accumulation in the brain (Morganti-Kossmann et al., 2002; Bao et al., 2012). Thus, here we observed by MPO activity that administration of FeTTPS significantly reduced the neutrophilic accumulation caused by TBI.

Moreover, iNOS production as well as plasma NO levels, evaluated as NO_2_/NO_3_, have been also involved in TBI (Orihara et al., 2001). iNOS expression was observed near inflammatory areas mainly in neutrophils/macrophages, while iNOS inhibition was protective against damage in TBI mice models (Khan et al., 2004). In the present study we demonstrated that FeTPPS treatment was able also to inhibit iNOS expression after injury, demonstrating the benefical role of FeTPPS against iNOS-mediated damage. Furthermore, during TBI the oxidative stress activate an important intracellular transduction system the Mitogen activated protein kinases (MAPK) pathway, specifically JNK, that are activated in response of oxidative stress, environmental stress and toxic insults, during pathological conditions (Benhar et al., 2001; LaChapelle et al., 2007). Thus in the present study we observed that MAPKs levels were significantly increased after TBI, whereas FeTPPS treatment prevented TBI-induced MAPPKs activation.

Apoptosis is another important regulator of brain injury controlling the degeneration of different cell types and involving oligodendrocytes and microglia (Mandai et al., 1997). In the present work, we studied the apoptotic transcriptional changes, through the pro-apoptotic Bax and the anti-apoptotic Bcl-2 proteins; we cleraly observed that FeTPPS treatment considerably reduced Bax expression, restoring the basal levels of Bcl-2.

In conclusion, we demonstrated that peroxynitrite production and inflammatory process are associated with brain damage aroused by trauma, contributing to secondary brain damage and progressive neuronal loss. Here, we report evidence that support the dual, the beneficial or the deleterious role of neuroinflammation after traumatic brain injury. Thus, the ONOO^−^ decomposition catalysts represent an innovative approach to supervise the pathological sequelae associated with TBI. To better understand the key role that peroxynitrite plays in the progress of inflammatory diseases, such as TBI, these catalysts were used as pharmacological tools *in vivo* models of human disease. Successively, this should provide more helpful treatment strategies for diseases in the clinic.

## Author contributions

SC and EE planned experiments and analyzed the results. GB and DI performed experiments and prepared the manuscript. MC and GC performed the biochemical analysis. RD performed histology and immunohistochemical staining. IP performed immunofluorescence staining. All authors read and approved the final manuscript.

### Conflict of interest statement

The authors declare that the research was conducted in the absence of any commercial or financial relationships that could be construed as a potential conflict of interest.
